# Combination Therapy with Oleanolic Acid and Metformin as a Synergistic Treatment for Diabetes

**DOI:** 10.1155/2015/973287

**Published:** 2015-02-18

**Authors:** Xue Wang, Yupeng Chen, Daoud Abdelkader, Waseem Hassan, Hongbin Sun, Jun Liu

**Affiliations:** ^1^National Drug Screening Center and Jiangsu Key Laboratory of TCM Evaluation and Translational Research, China Pharmaceutical University, 24 Tongjiaxiang, Nanjing 210009, China; ^2^Center for Drug Discovery, State Key Laboratory of Natural Medicines, China Pharmaceutical University, 24 Tongjiaxiang, Nanjing 210009, China

## Abstract

*Aims and Background*. Type 2 diabetes is a chronic disease that cannot be treated adequately using the known monotherapies, especially when the disease progresses to an advanced stage. In this study, we explore the possibility of treating the disease with a novel combination approach of oleanolic acid (OA), a glycogen phosphorylase (GP) inhibitor, and metformin. *Methods*. Db/db mice were randomly divided into four groups: a db/db control group, db/db mice treated with OA (250 mg/kg), db/db mice treated with metformin (100 mg/kg), and db/db mice treated with a combination of OA and metformin. All mice were treated for four weeks. The effects of the treatments on glucose homeostasis were measured using an OGTT, an assessment of insulin sensitivity and signaling in the liver, and the hepatic glucose production. *Results*. Combination therapy with OA and metformin significantly reduced the blood glucose and insulin levels and improved the liver pathology compared with that for the monotherapy in the db/db diabetic mouse model. We also found that the combination therapy significantly increased the mRNA expression of glycogen synthesis and decreased the GP, PGC-1*α*, PEPCK1, and G-6-Pase levels. In addition, the combination therapy with OA and metformin significantly increased the phosphorylation of AKT, PI3K, AMPK, and ACC and decreased the protein expression levels of G-6-Pase, PEPCK1, and TORC compared with those for either monotherapy. The combination therapy also reduced the phosphorylation of mTOR and CREB. *Conclusions*. Our results suggest that the combination therapy with OA and metformin has synergistic effects on the symptoms of db/db diabetic mice by improving glucose and insulin homeostasis.

## 1. Introduction

Diabetes mellitus and the associated microvascular and macrovascular complications are the major threat to diabetic patients. Numerous studies have clearly shown that effective blood glucose control is the key to preventing and treating diabetic complications [1–3]. In addition to insulin resistance, one of the major reasons for hyperglycemia in diabetic patients is increased hepatic glucose production. Suppressing hepatic glucose production is a viable approach for treating diabetes [4]. Because the balance between gluconeogenesis and glycogenolysis determines the hepatic glucose production, either of the pathways could be targeted to treat diabetes.

Metformin has been the first-line drug for treating diabetes for the past five decades. The major mechanism by which metformin lowers blood glucose levels is through the inhibition of hepatic gluconeogenesis [5]. We previously reported that pentacyclic triterpene inhibits glycogen phosphorylase (GP) and can be used as a new strategy to lower blood glucose [6] using natural substances with low toxicity [7–11].

Oleanolic acid (OA) is a natural component of many plant foods and medicinal herbs and also a pentacyclic triterpene that exerts beneficial effects against diabetes and metabolic syndrome [12–15]. OA is therapeutically effective without inducing significant side effects [16–18]. Our previous studies showed that OA can inhibit GP, and its hypoglycemic effect is partially mediated by the inhibition of glycogenolysis in vivo [17, 18].

The complicated pathogenesis of diabetes suggests that a combination therapy may be more effective in treating this disease. In this study, we test whether a combination therapy using OA and metformin would have synergistic effects on improving glucose metabolism in diabetic conditions.

## 2. Materials and Methods

### 2.1. Drugs and Chemicals

**Figure 1:**
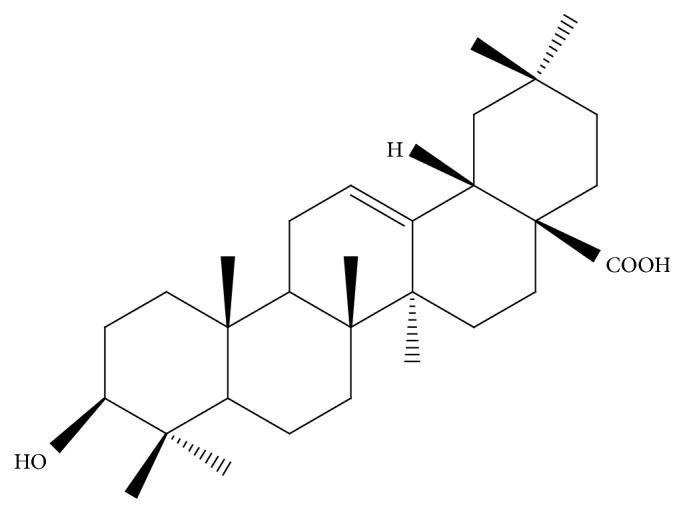
The structure of OA.

Oleanolic acid (structure shown in Figure 1) was obtained from the Center for Drug Discovery at China Pharmaceutical University. Metformin (purity > 98%) was obtained from Sigma (St. Louis, MO, USA). The mouse insulin enzyme immunoassay ELISA kit was purchased from Invitrogen. Serum triglyceride (TG), total cholesterol (TC), high-density lipoprotein cholesterol (HDL-C), and low-density lipoprotein cholesterol (LDL-C) kits were purchased from Whitman Biotech (Nanjing, Ltd., China). Antibody (Ab**) **for G-6-Pase was purchased from Santa Cruz Biotechnology (Santa Cruz, CA, USA). TORC2 was from Millipore (Billerica, MA, USA), and phospho-CREB and *β*-actin were from Abcam (Cambridge, MA, USA). The Abs against protein kinase B (PKB/Akt), phospho-Ser473-Akt (pAkt), PI3K, phospho-PI3K, AMPK, phosphor-AMPK, ACC, phosphor-ACC, PEPCK1, mTOR, and phospho-mTOR were purchased from Cell Signaling Technology (Beverly, MA, USA). The HRP-conjugated secondary Abs, affinity-purified mouse anti-rabbit IgG, and rabbit anti-mouse IgG were purchased from Sigma (St. Louis, MO, USA).

### 2.2. Animals and Treatments

Male C57BL/KsJ-Lepdb (db/db) mice were obtained from the Model Animal Research Center of Nanjing University, China. The animals were maintained in a 12 h light-dark cycle at a temperature of 25°C with free access to water and regular chow. To investigate the effect of OA and metformin, the seven-week-old db/db male mice were divided into four groups (*n* = 6/group). The mice were given vehicle (0.25% w/v CMC), OA (250 mg/kg), metformin (100 mg/kg), or a combination of the two drugs once daily by intragastric administration for 28 days. The body weight and food intake were recorded regularly. After 8 hours of fasting, blood glucose concentrations were monitored using tail vein blood and a glucometer (One-Touch Ultra, Lifescan) every week. The mice were fasted for 12 hours for an oral glucose tolerance test (OGTT, day 21) with the slight modifications described in the figure legend. Blood samples were obtained from the tail vein, and the serum insulin concentration was measured using an ELISA kit. At the end of the experiment (day 28), the mice were fasted for 8 hours, and blood samples were collected from the orbital venous plexus and centrifuged for serum separation. The animals were euthanized by CO_2_ inhalation, and the liver tissue was excised and stored in 4% paraformaldehyde for further analysis. All of the animal experiments were conducted according to the Guidelines of Experimental Animal Care issued by the Committee for the Purpose of Control and Supervision of Experiments on Animals.

### 2.3. Biochemical Parameter Analysis

At the end of the experiment, the blood glucose levels and the serum levels of triglyceride (TG), total cholesterol (TC), high-density lipoprotein cholesterol (HDL-C), and low-density lipoprotein cholesterol (LDL-C) were measured using commercially available kits following the manufacturer's protocol. The serum concentration of insulin was measured using ELISA following the protocol provided in the kit. The homeostatic model assessment (HOMA) value was calculated as described by Matthews et al. [19]. Insulin resistance was determined using the HOMA method and the following equation: HOMA value for insulin resistance (HOMA-IR) = fasting insulin (mU/mL) ∗ fasting glucose (mmol/L)/22.5. The liver index was calculated as follows and used to assess the organ health and hepatic histological damage: liver index = liver weight/body weight. To evaluate the glycogen content of the tissue, the method of Lillie and Greco was used [20]. Liver samples were homogenized in 50 mM Tris/HCl buffer at pH 7.4, containing 150 mM NaCl, 1 mM EDTA, and 1 mM PMSF. The liver triglyceride and total cholesterol contents were analyzed using the previously described protocol.

### 2.4. Hepatic Histology

The tissue was subsequently embedded in paraffin and sectioned to a thickness of 5 mm using a microtome. Tissue sections prepared on aminosilane-treated slides were deparaffinized and dehydrated using a graded alcohol series and distilled water. The tissue sections were stained with hematoxylin and eosin. The glycogen content was determined by comparative PAS staining with or without prior amylase treatment (Histoserv, Germantown, MD). For the detection of immunofluorescence, the slides were incubated with a mouse monoclonal *α*-actin antibody (1 : 1000; Santa Cruz Biotechnology, Inc.) together with a rabbit antibody for G-6-Pase (1 : 500; Santa Cruz Biotechnology, Inc.) and a rabbit antibody for PEPCK1 (Cell Signaling), followed by a TRITC-conjugated goat anti-mouse antibody and a FITC-conjugated goat anti-rabbit antibody (1 : 100; Huawei ImmunoResearch Laboratories Inc.). After thorough washing, the slides were mounted and visualized using a fluorescent microscope.

### 2.5. Western Blotting

Lysis buffer (25 mM Tris-HCl pH 7.5, 100 mM NaCl, 2.5 mM EDTA and EGTA, 20 mM NaF, 1 mM Na_3_VO_4_, 20 mM Na *β*-glycerophosphate, 10 mM Na pyrophosphate, 0.5% Triton X-100, 0.1% *β*-mercaptoethanol, and protease inhibitor cocktail (Roche)) was used to lyse the tissue. SDS-PAGE-resolved proteins were transferred to nitrocellulose membranes (GE Healthcare) and incubated with Abs to detect the G-6-Pase, TORC2, phospho-CREB, Akt, phospho-Ser473-Akt, PI3K, phospho-PI3K, AMPK, phosphor-AMPK, ACC, phosphor-ACC, PEPCK1, mTOR, and phospho-mTOR protein levels. After the incubation with the HRP-conjugated secondary Ab, the signals were detected using an ECL kit. All Western blot results are representative of at least two independent experiments.

### 2.6. RNA Extraction and Quantitative RT-PCR

Total RNA from the liver was extracted using TRIZOL Reagent (Invitrogen). The RNA concentration was determined using UV spectrophotometry. RT-PCR was performed using Thunderbird SYBR Master Mix (TAKARA, Japan). The PCR was performed on a Real-Time PCR Detection System (StepOnePlus, ABI) with the following cycles: 95°C for 1 min, followed by 40 cycles of 95°C for 15 s, 58°C for 15 s, and 72°C for 45 s to detect the GP, GS, PGC-1*α*, G-6-Pase, PEPCK1, and GLUT2 gene levels (sequences shown in Table 1). GAPDH expression was used as an internal control. The 2^−ΔΔCT^ was calculated for every sample and normalized to GAPDH. All RT-PCR results are representative of three independent experiments.

### 2.7. Statistical Analysis

Data are expressed as the mean ± SEM unless otherwise indicated. A two-tailed Student's *t*-test and one-way ANOVA were performed to test for statistically significant differences. A value of *P* < 0.05 was considered significant at a 95% confidence level.

## 3. Results

### 3.1. Effects of the Combined Treatment on Food Consumption, Body Weight, Liver Weight, and Serum Biochemical Parameters in db/db Mice

Vehicle (0.5% CMC-Na), OA (250 mg/kg), metformin (100 mg/kg), or a combination of the two drugs was intragastrically administered to 7-week-old obese db/db male mice once daily for 4 weeks. No significant alterations in the overall body weight gain, liver index, and liver biochemical parameters were found. However, significant differences in food intake, water intake, and the levels of serum biochemical parameters were observed (Table 2).

### 3.2. Effects of the Combined Treatment on the Fasting Serum Insulin (FSI) Level, Fasting Serum Glucose (FSG) Level, and Other Pharmacodynamics Biomarkers in db/db Mice

**Figure 2:**
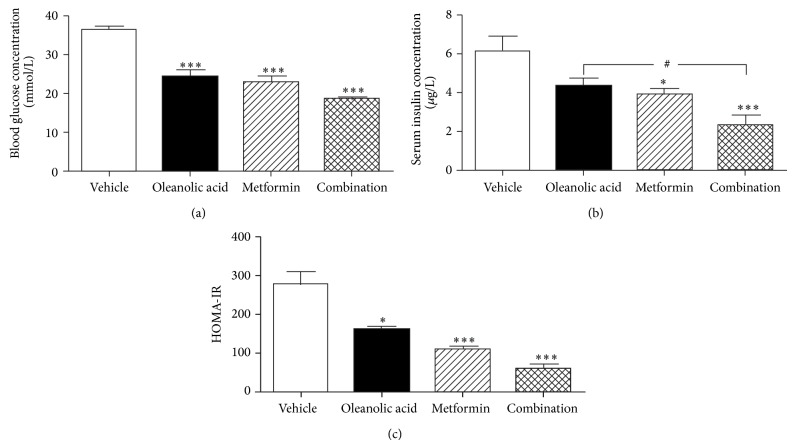
The effect of oleanolic acid, metformin, and their combination on the FSI level, FPG level, and HOMA-IR in db/db mice. (a) The FPG level was measured after 8 h of fasting in db/db mice treated with vehicle, oleanolic acid, metformin, or the combination for 4 weeks. (b) The FSI level was measured after 8 h of fasting in db/db mice treated with vehicle, oleanolic acid, metformin, or the combination for 4 weeks. (c) The HOMA-IR index was calculated for each group. Data are shown as the mean ± SEM. ^*^ *P* < 0.05, ^**^ *P* < 0.01, and ^***^ *P* < 0.001 indicate significant differences for comparisons with the vehicle group; ^#^ *P* < 0.05 indicates significant differences for comparisons with the monotherapy group (*n* = 6).

At baseline, the vehicle, OA, metformin, and combination groups were matched with respect to fasting blood glucose level. After four weeks of treatment, the fasting blood glucose level was significantly lower in the OA, metformin, and combination groups than in the vehicle group (Figure 2(a)). Importantly, the mice treated with the combination of OA and metformin achieved a more significant decrease in the fasting blood glucose level compared with that for the mice that received the monotherapies. Similarly, the fasting serum insulin levels were significantly decreased in the metformin group and combination group compared with that in the vehicle group (Figure 2(b)). In addition, the insulin sensitivity (as indicated by the insulin resistance index HOMA-IR) was also improved in the mice treated with either monotherapy or the combination therapy, and the greatest improvement occurred in the animals treated with the combination of the two agents (Figure 2(c)).

### 3.3. Effect of the OA, Metformin, and OA-Metformin Combination Therapy on the Oral Glucose Tolerance Test Results in db/db Mice

**Figure 3:**
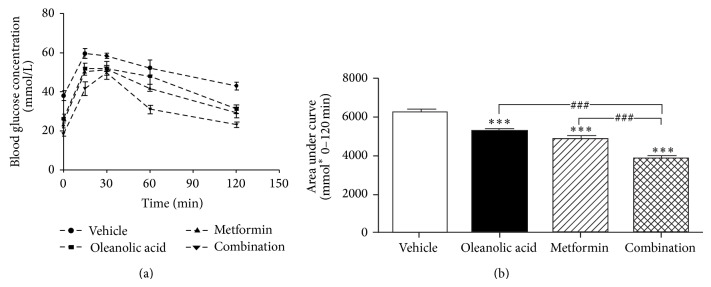
Chronic treatment results in normalized hyperglycemia and improved glucose tolerance in db/db mice. (a) Glucose tolerance test in oleanolic acid-, metformin-, and combination-treated and untreated db/db mice. After three weeks of treatment with OA, metformin, and the combination, mice were fasted for 12 hours and then given glucose (1.5 g/kg). The blood glucose levels in the tail tip blood were measured at 0, 15, 30, 60, and 120 minutes after glucose administration. (b) One-way ANOVA of the area under curve showed that the drugs can significantly improve glucose tolerance in db/db mice compared with that in untreated db/db mice. Data are shown as the mean ± SEM. ^*^ *P* < 0.05, ^**^ *P* < 0.01, and ^***^ *P* < 0.001 indicate significant differences for comparisons with the vehicle group; ^###^ *P* < 0.001 indicates significant differences for comparisons with the monotherapy group (*n* = 6).

To further evaluate the changes in glucose metabolism and insulin secretion in db/db mice, an OGTT was performed 3 weeks after the start of the treatments. Although the glucose levels during the OGTT in the OA-treated mice were similar to those of the metformin-treated mice (Figures 3(a) and 3(b)), the combination therapy-treated mice showed a marked decrease in blood glucose levels compared with those in the vehicle-treated group.

### 3.4. Liver Inflammation

**Figure 4:**
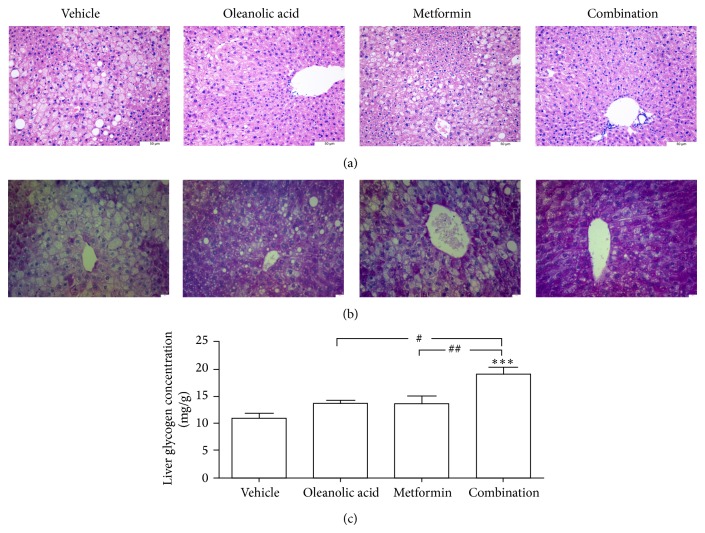
Histological analysis using H&E and PAS staining after 4 weeks of treatment with oleanolic acid, metformin, and the combination. (a) Representative photomicrographs depicting the H&E staining of liver sections in untreated and treated db/db mice. (b) Representative photomicrographs depicting the PAS staining of liver sections in untreated and treated db/db mice. (c) Liver glycogen. ^*^ *P* < 0.01 versus untreated db/db mice. ^***^ *P* < 0.001 versus untreated db/db mice. ^###^ *P* < 0.001 versus oleanolic acid-treated db/db mice. Each bar represents the mean ± SEM of the group (*n* = 6).

**Figure 5:**
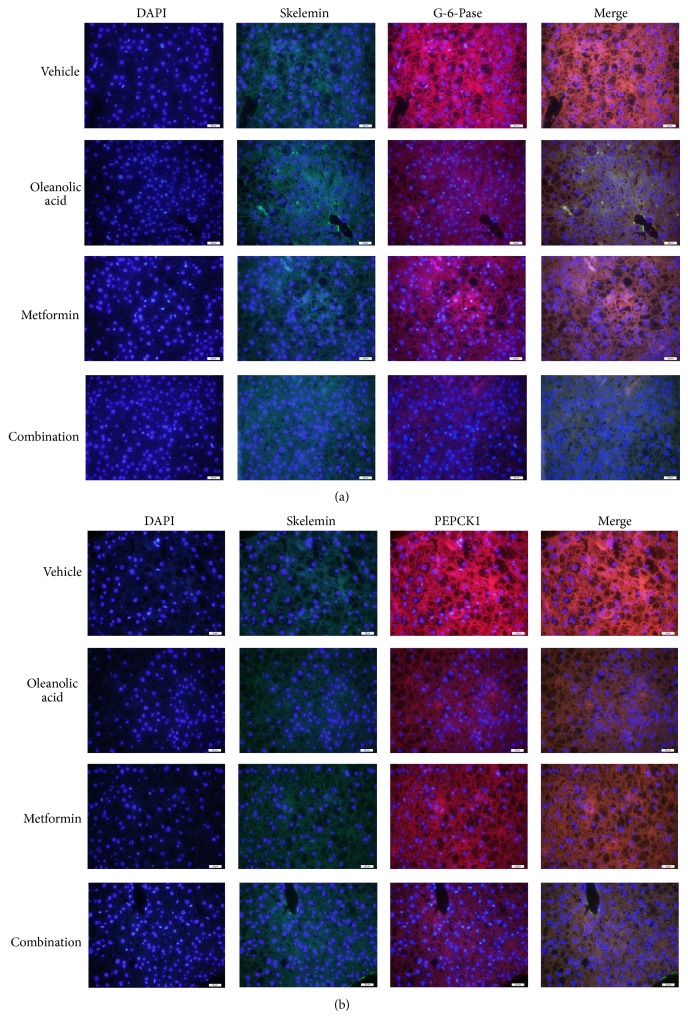
Histological analysis using immunofluorescence staining after 4 weeks of treatment with oleanolic acid, metformin, or the combination. (a) Double immunofluorescence of *α*-actin (green fluorescence) and G-6-Pase (red fluorescence) from the pancreas of different groups; merged figures are shown on the right. (b) Double immunofluorescence of *α*-actin (green fluorescence) and PEPCK1 (red fluorescence) from the pancreas of different groups; merged figures are shown on the right.

The H&E-stained liver samples obtained from the four groups showed signs of liver inflammation. Although the vehicle group showed lobular inflammation and hepatocyte ballooning, the OA monotherapy group showed slightly reduced levels of inflammatory cell infiltrates and hepatocyte degeneration. The metformin monotherapy group showed an even greater reduction in these parameters. Furthermore, the combination therapy group showed minimal inflammatory cell infiltrates and only a small amount of hepatocyte degeneration (Figure 4(a)). The PAS stain showed that the liver glycogen level in the combination therapy group was significantly higher than those in the vehicle group or the OA and metformin groups (Figure 4(b)). The liver glycogen content in the combination therapy group was significantly higher than that in the vehicle group and either monotherapy group (Figure 4(c)). As shown in Figures 5(a) and 5(b), the G-6-Pase and PEPCK1 immunoreactivity in liver was significantly decreased in the combination group compared with that in the vehicle group.

### 3.5. Effect of the OA, Metformin, and OA-Metformin Combination Therapy on Hepatic mRNA Expression Levels of Key Regulators Associated with Gluconeogenesis and Glycogenolysis

**Figure 6:**
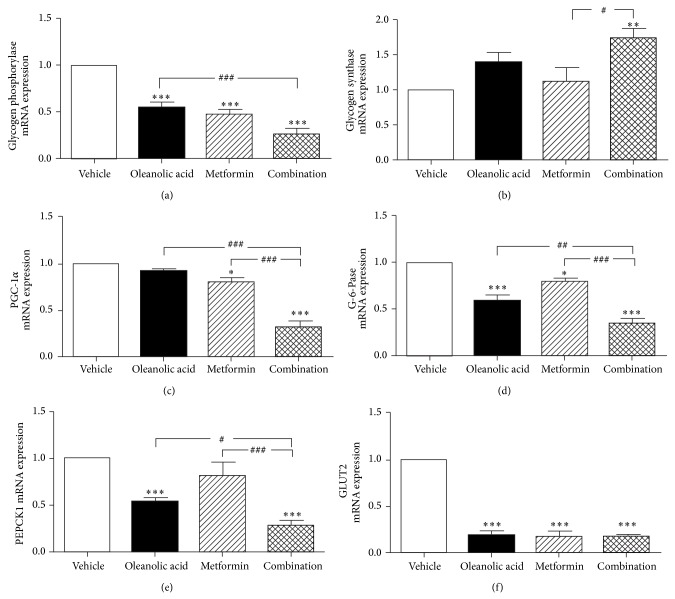
Analysis of the liver mRNA expression levels of markers associated with glucose metabolism.

In the present study, we also examined the effect of each treatment on the expression of several important genes for controlling hepatic gluconeogenesis and glycogenolysis, including PGC-1*α*, GP, G-6-Pase, and PEPCK1. As shown in Figure 6, the combination therapy with OA and metformin significantly inhibited the mRNA expression of PGC-1*α* and decreased the mRNA expression levels of GP, G-6-Pase, and PEPCK1. However, the treatment increased the GS mRNA expression level in db/db mice (Figure 6). These results demonstrate that, compared with the monotherapies, the combination therapy inhibits gluconeogenesis and glycogenolysis more extensively in db/db mice.

### 3.6. Liver Protein Expression Levels of Markers Associated with Gluconeogenesis and Glycogenolysis

**Figure 7:**
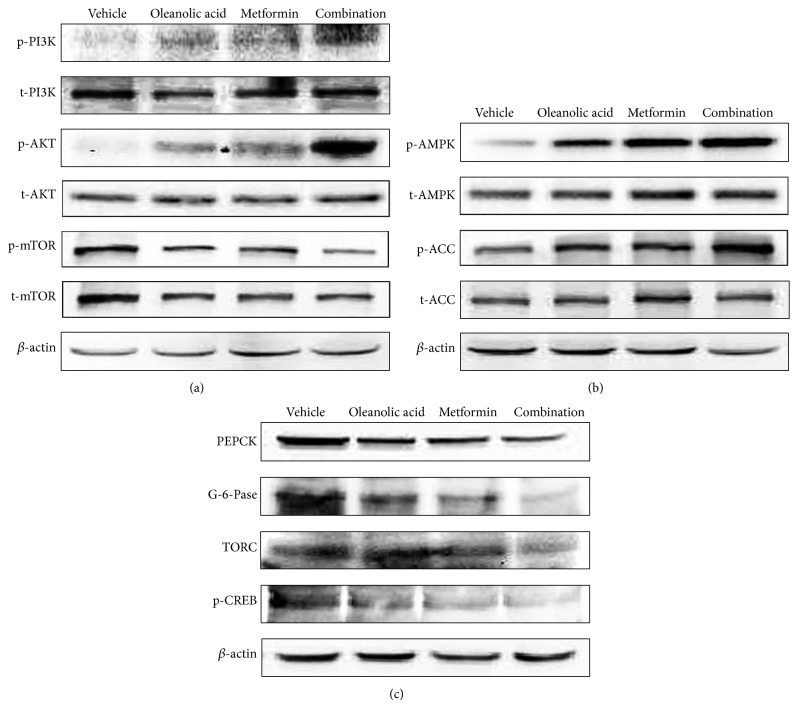
Analysis of the liver protein expression levels of markers associated with glucose metabolism. (a) Effect of OA, metformin, and the combination on the insulin-stimulated phosphorylation of Akt in mouse livers. After the experiment, the livers were harvested, and the phosphorylation of Akt, PI3K, and mTOR was measured using Western blotting. (b) Effect of OA, metformin, and the combination on the phosphorylation of AMPK and PCC in db/db mouse livers was evaluated using Western blotting. After the experiment, the livers were harvested, and the Akt phosphorylation was measured using Western blotting. (c) Effect of OA, metformin, and the combination on gluconeogenesis in mouse livers. The protein expression of PEPCK1, G-6-Pase, TORC, and phospho-CREB was evaluated using Western blotting.

We have also examined the effects of the OA, metformin, and combination therapy on the insulin signaling pathway induced phosphorylation of PI3K, Akt, and mTOR and on the AMPK pathway (Figure 7(a)). These treatment groups also showed activation of AMPK phosphorylation compared with that in the db/db control diabetic group. This phosphorylation can also stimulate the downstream phosphorylation of ACC (Figure 7(b)). We investigated whether OA, metformin, or the combination therapy inhibited gluconeogenesis by reducing G-6-Pase, PEPCK1, TORC, and p-CREB. The protein levels of TORC and p-CREB were increased in the db/db mice. The administration of OA, metformin, or a combination of the two drugs lowered the protein levels of TORC and p-CREB. Furthermore, the protein expression of G-6-Pase and PEPCK1 decreased (Figure 7(c)).

## 4. Discussion

The current study investigated a therapy that combined OA and metformin as a synergistic treatment for diabetes mellitus and attempted to evaluate the underlying mechanisms. The combination therapy (OA and metformin) was found to possess different but complementary mechanisms of action on the plasma glucose and insulin levels that prevented the development of diabetes. In addition to the improved liver pathology, the combination therapy potently decreased the mRNA expression levels of GP, PGC-1*α*, PEPCK1, and G-6-Pase and increased the synthesis of glycogen. Interestingly, several of these genes showed statistically significant additive or synergistic changes. We also found that the combination therapy could partly activate the AMPK and AKT signaling pathway to a greater extent compared with the activation by either of the monotherapies.

Metformin is a widely used hypoglycemic agent for the treatment of diabetes, with a well-known ability to reduce hepatic gluconeogenesis. At the hepatocellular level, metformin is known to improve insulin-mediated glycogen synthesis and inhibit gluconeogenesis [21]. As a natural triterpenoid, OA has been used in traditional Chinese medicine to treat hepatitis since the 1970s. In recent years, OA has been reported to effectively lower blood glucose levels by promoting insulin signal transduction [22, 23]. In addition, Ngubane et al. [24] reported that OA restored depleted glycogen levels and thus provided another approach for treating diabetes. Due to their different mechanisms of action, the administration of metformin and OA together appears to be an effective strategy for lowering blood glucose in a synergistic manner.

To gain the deeper insight into diabetes mellitus, several animal models have been developed in the last several decades; these models include the streptozotocin-induced diabetic rat model and conventional or genetically modified models [25]. The db/db mouse has been the most commonly employed model that has been used to investigate the pathogenesis of diabetes mellitus in many studies since 1966 [26]. The db/db mouse model, which is characterized by deficient leptin receptor activity, is known to cause deleterious changes in various tissues triggered by hyperglycemia, dyslipidemia, obesity, insulin resistance, and advanced glycation [27]. Among these pathogenic factors in diabetes, hyperinsulinemia and insulin resistance are associated with diabetes risk, and increases in these particular factors have been shown in subjects that have a metabolic abnormality [28, 29].

The mice under this model have a clear obese phenotype, with enhanced food intake and increased fasting blood glucose and lipid levels after 7 weeks; afterwards, the symptoms gradually worsen to become severe diabetic complications. The results clearly showed a marked reduction in water intake in the mice that only received OA, and the combination therapy group had a particularly significant change in food and water intake compared with that in the monotherapy groups. This finding indicated that OA could partly improve energy metabolism disorders and maintain body stability. The changes in the food and water intake might be interpreted as a reflex biological effect that is induced by the combination therapy, or the changes might be a compensatory response to the level of some other parameter. We also found that either the metformin or OA monotherapy tended to attenuate diabetic symptoms; however, the combination therapy using both compounds exerted synergistic effects on lipid and glucose metabolism. Compared with the monotherapy, the combination therapy clearly inhibited the elevated lipid levels induced by the db/db mouse model. Metformin had only a slight advantage in reducing triglycerides and total and HDL-cholesterol levels compared with the reduction by the OA treatment, but the combination therapy showed an extremely potent synergistic action. Significant improvements were also observed in the plasma glucose and insulin levels and in the homeostasis model assessment-insulin resistance (HOMA-IR) index for the combination therapy group. The attenuation of diabetic pathology characterized by a reduction in the serum glucose levels and an improvement in insulin resistance could possibly result from the synergistic effects by which metformin and OA inhibit both gluconeogenesis and glycogenolysis. We demonstrated the therapy combining OA and metformin exerted favorable effects; the improvement in hyperglycemia and lipid metabolism induced by the combination therapy was greater than that for either of the monotherapies, or the combination treatment was successful in this spontaneous animal model of diabetes mellitus.

Liver glycogen is synthesized in response to elevated concentrations of glucose and insulin [30]. Many genes play a crucial role in the process of hepatic glycogen synthesis. Glycogen phosphorylase (GP) is the enzyme responsible for the synthesis of glucose-1-phosphate, the source of energy for muscles and the rest of the body [31]. G-6-Pase catalyzes the hydrolysis of glucose 6-phosphate (G6P) to glucose and inorganic phosphate [32]. Our results suggest that the treatment groups, especially the combination therapy group, had fewer droplets than the number of droplets in the control group. The combination therapy significantly improved the glycogen content in the liver compared with that in either of the monotherapy groups. Signaling pathways were studied, and the combination therapy effectively reduced hepatic gluconeogenesis by decreasing the mRNA expression level of PGC-1*α*, G-6-Pase, and PEPCK. In addition, the combination therapy significantly inhibited the mRNA levels of GP and GLUT2 and increased the mRNA level of GS, thus decreasing hepatic glucose production.

AMPK is a heterotrimer composed of alpha-catalytic and beta- and gamma-regulatory subunits; some genes in this signaling pathway are subject to alternative splicing, which increases the range of possible heterotrimer combinations [33, 34]. Cellular stresses that inhibit ATP production or increase its consumption change the AMP : ATP ratio and activate the pathway [35]. A well-known role of AMP kinase (AMPK) is its participation in the regulation of many metabolic processes, including glucose uptake and fatty acid oxidation in muscle and fatty acid synthesis and gluconeogenesis in the liver. Due to its roles in energy regulation, the AMPK pathway is regarded as a potential therapeutic target for obesity and type 2 diabetes. In this study, we found that OA and metformin induced AMPK and ACC phosphorylation. Metformin and OA induced AMPK and ACC activation, reduced the downregulation of lipogenic enzymes, such as FAS, and increased pyruvate kinase activity, thus improving insulin resistance. The activation of AMPK inhibited both the transcription and translocation of PEPCK1 and G-6-Pase, resulting in an increase in insulin-stimulated glucose uptake. In addition, each of the monotherapies negatively regulated several proteins that are central to ATP-consuming processes, such as processes that involve p-CREB and TORC2, resulting in the downregulation or inhibition of gluconeogenesis. As expected, the combination therapy group significantly activated the phosphorylation of AMPK and ACC, thus improving the metabolic outcomes. Meanwhile, the group that received the combination treatment had lower G-6-Pase, PEPCK1, TORC, and p-CREB levels compared with those in the diabetic control group or either monotherapy group. Overall, these effects reduce gluconeogenesis.

Akt is essential for insulin stimulating events, such as glucose uptake and glycogen synthesis [36]. Our results suggest that the combination therapy stimulates PI3K, which phosphorylates AKT and downregulates p-mTOR to improve insulin resistance. mTOR is the target of both components of the combination therapy and connects the AMPK and AKT pathways [37, 38], and mTOR might be the key that induced the difference in action.

**Figure 8:**
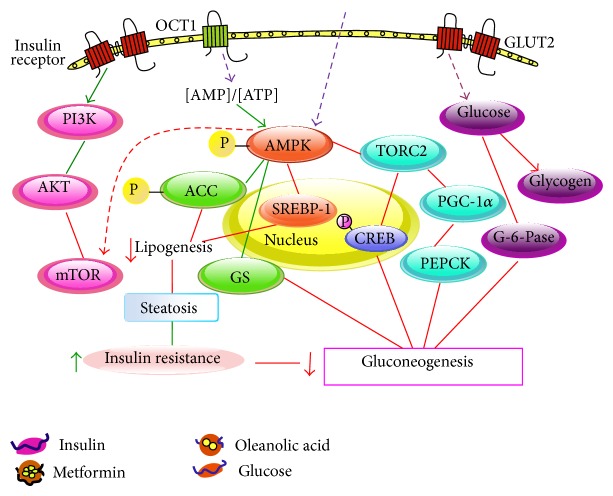
Proposed mechanism by which oleanolic acid and metformin inhibit diabetes.

In conclusion, the present study shows the efficacy of a combination therapy using OA and metformin; this combination controls hypoglycemia by reducing both gluconeogenesis and glycogenolysis. In addition to reducing blood glucose, this therapeutic approach has a key advantage in its modulation of glycogen synthesis, which may also have preventive and therapeutic benefits against diabetes. The combination therapy-treated group showed a markedly enhanced phosphorylation of AMPK and AKT, and the treatment induced decreased expression of proteins and genes that are relevant to glucose metabolism. Thus, our combination therapy results in a net inhibition of gluconeogenesis and glycogenolysis and reduces hepatic glucose production (Figure 8); these effects show its interesting potential as a treatment for diabetes.

## Figures and Tables

**Table 1:** Sequences of primers for the genes.

Gene	Sense primer (5′ to 3′)	Antisense primer (5′ to 3′)
PGC-1*α*	CAACAGCAAAAGCCACAAAG	GGGGTCAGAGGAAGAGATAA
PEPCK1	GAACAAGGAGTGGAGACC	TTCATAGACAAGGGGGAC
G-6-Pase	GTGGGCATCAATCTCCTC	GACTTCCTGGTCCGGTCT
GP	TAAAAAAGAACTTCAACCGC	TGTAGAACTCCAAAGACAGG
GS	CTCTGTGTCCTCGCTTCCA	GTCACCTTCGCCTTCGTCT
GLUT2	ATGAAGAAAGAAAAGGAAGA	TGCTGGTTGAATAGTAAAAT
GAPDH	CATGTTCGTCATGGGTGTGAAC	CAGTCTTCTGGGTGGCAGTGAT

**Table 2:** Comparison of characteristics of db/db mice with or without OA and/or metformin treatment.

Parameters	Vehicle group	Oleanolic acid (100 mg/kg)	Metformin (250 mg/kg)	Combination (350 mg/kg)
Weight gain (g)	4.13 ± 2.32	5.92 ± 1.98	5.60 ± 1.84	6.66 ± 1.59
Food intake (g/mouse)	7.49 ± 0.59	6.42 ± 0.45	6.03 ± 1.35	5.70 ± 1.33^*^
Water intake (mL/mouse/day)	2.85 ± 0.31	1.86 ± 0.52^*^	2.08 ± 0.47	1.66 ± 0.16^*^
Serum lipid (mmol/L)				
Triglyceride	1.96 ± 0.45	1.34 ± 0.31	1.34 ± 0.39^*^	1.12 ± 0.15^***^
Total cholesterol	5.59 ± 0.43	5.45 ± 0.25	4.51 ± 0.38^*^	3.64 ± 0.49^∗∗∗,###,Δ^
HDL-cholesterol	9.02 ± 0.97	7.54 ± 0.82^**^	5.87 ± 0.74^**^	5.01 ± 0.72^∗∗∗,##^
LDL-cholesterol	1.93 ± 0.34	1.61 ± 0.54	1.03 ± 0.28^*^	0.95 ± 0.14^**^
Liver changes				
Liver weight (g)	1.72 ± 0.23	1.58 ± 0.25	1.74 ± 0.37	1.57 ± 0.14
Liver index	0.0417 ± 0.0033	0.0389 ± 0.0039	0.0407 ± 0.0060	0.0408 ± 0.0030
Liver triglyceride (mg/g)	2.51 ± 0.46	2.35 ± 0.34	2.05 ± 0.36	1.91 ± 0.19
Liver total cholesterol (mg/g)	0.1012 ± 0.0507	0.0904 ± 0.0282	0.0727 ± 0.0229	0.0730 ± 0.0264

Data are expressed as the mean ± SEM. ^*^ *P* < 0.05, ^**^ *P* < 0.01, and ^***^ *P* < 0.001 indicate significant differences for comparisons with the vehicle group; ^##^ *P* < 0.01 and  ^###^ *P* < 0.001 indicate significant differences for comparisons with the OA monotherapy group, and  ^Δ^ *P* < 0.05 indicates significant differences for comparisons with the metformin monotherapy group.

## Abbreviations

GS: Glycogen synthase
GP: Glycogen phosphorylase
PEPCK1: Phosphoenolpyruvate carboxykinase 1
G-6-Pase: Glucose-6-phosphatase
GLUT2: Glucose transporter type 2
PGC-1*α*: PPAR*γ* coactivator 1*α*
AMPK: AMP-activated protein kinase
ACC: Acetyl-CoA carboxylase
TORC: Transducer of regulated CREB
mTOR: Mammalian target of rapamycin
CREB: cAMP-response element-binding protein.
